# LAD1 expression is associated with the metastatic potential of colorectal cancer cells

**DOI:** 10.1186/s12885-020-07660-0

**Published:** 2020-12-02

**Authors:** Byul Moon, Suk-Jin Yang, Seong Min Park, Sang-Hyun Lee, Kyu Sang Song, Eun-Jeong Jeong, Mijin Park, Jang-Seong Kim, Young Il Yeom, Jung-Ae Kim

**Affiliations:** 1 Personalized Genomic Medicine Research Center, Korea Research Institute of Bioscience and Biotechnology, Daejeon, 34141 South Korea; 2 Department of Functional Genomics, KRIBB School of Bioscience, University of Science and Technology, Daejeon, 34113 South Korea; 3 Present address: Department of Molecular and Human Genetics, Baylor College of Medicine, Houston, TX 77030 USA; 4 Present address: Translational Research Branch, Research Institute, National Cancer Center, Goyang, 10408 South Korea; 5 Biotherapeutics Translational Research Center, Korea Research Institute of Bioscience and Biotechnology, Daejeon, 34141 South Korea; 6 Department of Pathology, Chungnam National University College of Medicine, Daejeon, 34134 South Korea; 7 Department of Biological Science, College of Natural Sciences, Wonkwang University, Iksan, 54538 South Korea

**Keywords:** Ladinin-1, Colorectal cancer, Metastasis, Migration, Invasion

## Abstract

**Background:**

Anchoring filament protein ladinin-1 (LAD1) was related to the aggressive progression of breast, lung, laryngeal and thyroid cancers. However, the association of LAD1 with colorectal cancer remained unknown. Here, to determine the relationship of LAD1 with colorectal cancer progression, we explored the effect of LAD1 loss on the malignant features of colorectal cancer cells.

**Methods:**

We constructed LAD1-depleted cell lines and examined the effect of LAD1 deficiency on the phenotypic and molecular features of colorectal cancer cells in vitro. The function of LAD1 in metastasis in vivo was examined by establishing a spleen-to-liver metastasis mouse model. LAD1 protein expression in colorectal cancer patient specimens was assessed by immunohistochemistry of tumor microarrays.

**Results:**

We found that LAD1 was abundant in most colorectal cancer cells. In addition, high expression of LAD1 significantly correlated with poor patient outcome. LAD1 depletion inhibited the migration and invasion of two different colorectal cancer cell lines, SW620 and Caco-2, without affecting their proliferation. In addition, LAD1 loss led to defects in liver metastasis of SW620 cells in the mouse model. Immunohistochemistry of colorectal cancer tissues revealed LAD1 enrichment in metastatic tissues compared to that in primary tumor and normal tissues.

**Conclusion:**

These results suggest that LAD1 expression is associated with the metastatic progression of colorectal cancer by promoting the migration and invasion of cancer cells.

**Supplementary Information:**

The online version contains supplementary material available at 10.1186/s12885-020-07660-0.

## Background

Colorectal cancer is one of the deadliest cancers, accounting for approximately 10% of cancer-related deaths [1]. While the survival rates of colorectal cancer patients with localized and regional tumors are almost 89 and 79%, respectively, those with distant metastatic tumors are lower than 15%, which leads to the high mortality of this cancer type [2]. Treatment options for metastatic colorectal cancer are limited. While local therapies such as surgery of metastatic sites or radiofrequency ablation have mitigated the morbidity of patients, in many cases including metastasis with multiple sites in the body, the patients are not eligible for these options and are thus treated with systemic therapy [1]. For the last two decades, extensive efforts to develop better therapeutic strategies have increased the overall survival of metastatic cancer patients from 12 to nearly 30 months [3]. However, the short duration of overall survival, 30 months, still demands a better understanding of metastatic colorectal cancer to develop more efficacious diagnostic/prognostic factors and thus further improve patient prognosis.

LAD1 (ladinin-1) is a protein originally known as a collagenous anchoring filament protein of basement membranes in mammalian epidermal cells [4, 5]. However, a more recent study found cytosolic localization of LAD1 in mammary epithelial cells [6]. Moreover, LAD1 has been implicated in the progression of different cancers. Comparative proteomic analyses found that LAD1 is abundant in lung adenocarcinoma but not in normal lung tissue and benign lung nodules [7]. Proteomic profiling of laryngeal cancer tissues showed that LAD1 protein is enriched specifically in metastatic tissues but not in paired adjacent normal tissues and primary tumor tissues [8]. Moreover, analysis in METABRIC, a large clinical dataset of approximately 2000 breast cancer patients [9], showed that a high abundance of LAD1 transcripts is associated with poor prognosis in breast cancer patients [6]. A study of mouse thyroid cancer models revealed a molecular link between LAD1 expression and oncogenic signaling pathways in cancer by showing that the BRAFV600E mutation induced the expression of LAD1 transcripts [10]. Similarly, compared with normal tissues, the expression of the LAD1 transcript was elevated in human thyroid cancers carrying oncogenic mutations in genes such as BRAF, RET and RAS [10]. This finding suggests that different oncogenic signaling pathways upregulate LAD1 expression. Collectively, these studies demonstrate that aggressive progression of various cancers is accompanied by upregulation of LAD1 expression. Nevertheless, the molecular function of LAD1 in the progression of these cancers remains unclear. In addition, studies assessing the involvement of LAD1 in colorectal cancer progression are lacking.

In this study, we aimed to explore the expression of LAD1 and its functional involvement in colorectal cancer progression by using cell-based systems and a xenograft mouse model. Furthermore, we assessed the clinical relevance of LAD1 expression by examining public databases and patient tissue arrays.

## Methods

### Survival analyses

Survival analyses of data extracted from GSE14333 and GSE24549 were performed by survival analysis in the R package. Patients were stratified into two groups: high LAD1 expression (high) and low LAD1 expression (low) based on the median expression value. The log-rank test was used to compare the survival rates, and Cox proportional hazard regression analysis was carried out to determine the hazard ratios.

### Gene ontology analyses

To identify biological processes and pathways related to LAD1 expression in colorectal cancer, we performed Gene Ontology analyses with a list of genes that positively correlated with LAD1 expression. Positively correlated genes to LAD1 (R > 0.4, *p*-value< 0.001; R: correlation coefficient) were extracted from GSE14333 and GSE24549 using R packages. Finally, Gene Ontology analyses were performed with DAVID tools (DAVID Bioinformatics Resources 6.8).

### Cell cultures

The human colorectal cancer cell lines SW480, SW620, Caco-2, HT-29 and DLD-1 were purchased from ATCC (American Type Culture Collection) and maintained in DMEM (Welgene, LM011–05) and RPMI (Welgene, LM011–01) supplemented with 10% FBS (Gibco, 16000–044) and 1% antibiotics (Gibco, 15240–062). All of the indicated cell lines were cultured at 37 °C in a humidified atmosphere with 5% CO_2_ (Heraeus BB15 CO2 Incubator, Thermo Fisher). All the cell lines used in this study were authenticated and validated using STR genotyping performed by HPBIO, Inc. (Seoul, South Korea). Results of STR genotyping were provided in supplemental Table 3.

### Antibodies

The following antibodies were purchased from commercial suppliers: anti-LAD1 (Novus Biologicals, NBP1–86126), anti-E-cadherin (Santa Cruz, sc-7870), anti-N-cadherin (Santa Cruz, sc-7939), anti-vimentin (Santa Cruz, sc-32,322), β-actin (Santa Cruz, sc-47,778), α-tubulin (Santa Cruz, sc-23,948), anti-mouse and rabbit secondary peroxidase-conjugated antibodies (Millipore, AP124P and AP132P), DAPI (Invitrogen, 62,248), Alexa594-conjugated phalloidin (Invitrogen, A12381), anti-rabbit secondary antibody Alexa488 conjugate (Invitrogen, A-11034) and anti-Ki67 (Abcam, ab16667).

### RNA interference

siRNAs against LAD1 (si-LAD1#1-sense: 5′-CAGUGAAGUUGGGAGAGAA-3′; si-LAD1#2-sense: 5′-CAGACAACACAGUGAAGUU-3′) and negative control siRNA (sense: 5′-CCUACGCCACCAAUUUCGU-3′, Bioneer, SN-1003) were purchased from Bioneer. Human colorectal cancer cell lines were seeded in 6-well plates at 3X10^5^ cells per well, and then, 25 nM siRNAs were transfected for 48 h using RNAiMAX reagents (Invitrogen,13,778–150).

### Plasmid transfection

For transient plasmid transfection, SW480 cells were seeded at 2.5X10^5^ cells in 6-well plates. Next day, pCMV6-XL5-LAD1 (ORIGEN, LAD1 Human Untagged Clone, SC116651) was transfected with lipofectamine 3000 (Invitrogen, L3000–015) for 48 h. After transfection, cells that overexpressed LAD1 were used for Transwell assay.

### RNA extraction and reverse transcription (RT)-PCR analysis

Total RNA was extracted using TRIzol (Ambion, TRI Reagent Solution). cDNA was synthesized using the extracted total RNA, oligo dT (Bioneer, N-7503), dNTP mixture (Enzynomics, N002) and reverse transcriptase (Invitrogen, RevertAid reverse transcriptase, EP0442). RT-PCR and quantitative Real-Time PCR (qRT-PCR) were performed to analyze gene expression using synthesized cDNA and gene-specific primers (Bioneer). For RT-PCR, cDNA was amplified with gene-specific primers and PCR master mixture (Bioneer, AccuPower PCR Master Mix) by PCR machinery (MiniAmp Plus Thermal Cycler, Thermo fisher). The PCR products were analyzed via electrophoresis on agarose gel. qRT-PCR was carried out using a CFX96 Real-Time PCR system (Bio-Rad). qPCR reactions were prepared in mixtures with cDNA template, 300 nM forward and reverse primers and ready-to-use reaction master mix (Bio-Rad, iQ SYBR Green Supermix). The conditions of thermal cycling were as follows: initial incubation at 95 °C for 5 min; 45 cycles of 95 °C for 10s, annealing at 60 °C for 30s and extension at 72 °C for 30s. mRNA gene expression was normalized to β-Actin (ACTB) mRNA. Supplemental Table 1 provides the primer sequences used for RT-PCR.

### Protein extraction and western blot

Whole-cell lysates were prepared by cold RIPA buffer (50 mM Tris-HCl, pH 8.0, 150 mM NaCl, 1% Triton X-100, 0.5% sodium deoxycholate, and 0.1% SDS). Proteins were quantified with the Bradford assay, and equal amounts of proteins were used for each sample. To resolve by SDS-PAGE, 5X SDS sample buffer (250 mM Tris-HCl, pH 6.8, 10% SDS, 50% glycerol, 0.5 M dithiothreitol, and 0.25% bromophenol) was added to cell lysates and boiled at 100 °C for 10 min. After SDS-PAGE, proteins were transferred to nitrocellulose membranes (PALL, Biotrace™NT), and membranes were blocked using 5% skim milk in TBST buffer. Primary antibodies incubated with membranes and HRP-conjugated secondary antibody were used to detect chemiluminescent signals from specific target proteins. The immunoblotting signals were visualized by using LAS3000 (Luminescent Image analyzer, FUGIFILM).

### Cell viability assay

For the cell viability assay, LAD1 knockdown cells (2X10^3^ cells per well) were seeded into black 96-well plates (Corning, 3603). After 72 h, 20 μl of CellTiter-Blue Reagent (Promega, CellTiter-Blue® Cell viability assay) was added to each well and incubated for 2 h. The fluorescence signal at Ex/Em = 530/590 nm was detected by a fluorometer (SYNERGY/HTS, BioTek).

### Transwell assay

Transwell inserts (Corning, 3422) were precoated with human collagen-type IV for 1 h. Specifically, for the invasion assay, the upper chamber was coated with Matrigel (Corning, 354,234) for 1 h. Cells were suspended in serum-free DMEM, and 5X10^4^–1X10^5^ cells were seeded in each well containing DMEM with 10% FBS as previously described [11]. After incubation for 24 h, the migrated or invaded cells were stained with 0.5% crystal violet solution. Cells that had migrated or invaded were counted manually (200x magnification field, 5 field per replicate).

### Invadopodia formation assay

A QCM™ gelatin invadopodia assay (EMD Millipore Corp, ECM670) was used to evaluate invadopodia formation in SW620 cells transiently knocked down with siRNA. In detail, poly-L-lysine was diluted in distilled water to coat 8-well glass chamber slides (Thermo Scientific, Lab-Tek chamber slide, 177,402). After a PBS wash, glutaraldehyde was added to each well for 15 min to activate the poly-L-lysine surface. Fluorescently labeled gelatin was coated onto each well for 10 min and disinfected with 70% ethanol. Cells at approximately 50% density (2X10^4^ cells per the condition) were seeded in each well and incubated in previously indicated cell culture conditions for 48 h before taking fluorescent images with a fluorescence microscope (Inverted Microscope Eclipse Ti-S, Nikon).

### Immunofluorescence

Immunofluorescence staining was performed as described previously [12]. Briefly, cells were seeded at 1X10^4^ cells on precoated glass plates (Ibidi, 89,626) and fixed in 4% PFA and permeabilized using 0.3% Triton X-100 in PBS for 10 min. After blocking with 1% bovine serum albumin in PBST, the primary antibody specific for LAD1 was incubated, and then Alexa 488-conjugated goat anti-rabbit antibody was incubated for 1 h. Finally, Alexa594-conjugated phalloidin and DAPI were used to stain actin and the nucleus. Fluorescent images were analyzed by a fluorescence microscope (Inverted Microscope Eclipse Ti-S, Nikon).

### Lentivirus production and constructing stable cell lines

For in vivo experiment, SW620 cells stably expressing shSCR and shLAD1 RNAs were constructed as described below. Lentivirus expressing shSCR and shLAD1 RNAs were prepared from 293 T cells by co-transfection with shRNA plasmid (shSCR: SHC002, MISSION pLKO.1-puro, CCGGCAACAAGATGAAGAGCACCAACTCGAGTTGGTGCTCTTCATCTTGTTGTTTTT; shLAD1–1: TRCN0000077998, CCGGGTGTGCAACCACTTACCCTTTCTCGAGAAAGGGTAAGTGGTTGCACACTTTTTG), psPAX2 and pMD2.G using lipofectamine reagents (Invitrogen, 18,324–012). After 48 h, lentiviral-supernatant was harvested and filtered with 0.45uM syringe filters (ADVANTEC, 25CS045AS). SW620 cells were infected with each lentivirus for 12 h and stabilized for another 1 day, and then puromycin selection was conducted for 72 h as described previously [11].

### Animal model of metastasis

Female CAnN.Cg-Foxn1nu/CrlOri (BALB/C-nu) mice (4 weeks old, 12-14 g) were purchased from OrientBio (Seongnam-si, Gyeonggi-do, South Korea). Mice were housed (specific pathogen free, individually ventilated cages with ad libitum feeding, wood bedding, 3 mice/cage) for 4 weeks prior to intrasplenic injection of SW620 cells expressing shSCR or shLAD1 RNAs. Cages with bedding, rodent diet (Harlan Teklad, 2018, sterilized) and autoclaved water were replaced twice a week during a whole period of animal experiment. Daily light and dark cycles (12-h light and 12-h darkness) were maintained with constant physiological conditions (temperature, 20 and 26 °C; humidity, 40–60%). Mice were randomly assigned into two groups before intrasplenic injection. Inhaled anesthesia was performed with 2% isoflurane with oxygen. A total of 2X10^6^ cells were resuspended in 50 μl of HBSS media and injected into the spleen via a 30-gauge needle. Two minutes after the injection, the splenic vessels were tied off with a suture. After surgery, mice were recovered under heating lamp for a while. Health status (body weight; recorded once a week, body temperature, activity, and behavior during handling) of mice were daily monitored until termination of the experiment. Mice were sacrificed by cervical dislocation 7 weeks after intrasplenic injection, and the liver was excised (*n* = 3 per group). The liver was examined by the naked eye to count metastatic nodules, and liver lobes were fixed in 10% formalin. This animal study was approved by Korea Research Institute of Bioscience & Biotechnology-IACUC (Daejeon, South Korea).

### Immunohistochemistry

Paraffin-embedded tumor-bearing liver sections were stained with hematoxylin and eosin (H&E) and an anti-Ki67 antibody [11–13]. Histology of liver-containing metastatic nodules was analyzed by H&E staining, and proliferation of tumors in each sample was analyzed by labeling with the anti-Ki67 antibody. Slide sections were deparaffinized by xylene and rehydrated with ethanol. Antigen retrieval was performed with sodium citrate buffer, pH 6.0, as recommended in the datasheet of the primary antibody. After the blocking step with goat serum in PBS, the primary antibody was incubated overnight at 4 °C. Endogenous peroxidase was blocked by 3% H_2_O_2_ incubation, and then slides were incubated with biotinylated goat anti-rabbit antibody. For the visualization of staining, DAB (Vector Laboratories, Inc., SK-4100) was used as a chromogen. Finally, slides were counterstained with hematoxylin.

### Human colon cancer tissue array staining

A human colon cancer tissue array slide (CDA3-G) was purchased from SuperBioChips (Seoul, South Korea). The tissue array slide contained 55 samples of colon cancer and normal tissue specimens. In addition, 9 samples were matched primary and metastasis tissues from different organs. Supplemental Table 2 included details of metastasis tissues. Immunohistochemistry of the tissue array slide was performed as previously indicated. The staining results of the tissue array were empirically graded by a pathologist.

### Statistical analysis

All data are presented as means of three independent experiments ± standard deviation. GraphPad Prism 7 software (GraphPad Software, Inc.) were used for statistical analyses. Student’s t-tests (two-tailed, unpaired) for two group comparisons and one-way ANOVA followed by Tukey’s post hoc test for more than two group comparisons were used to determine significant differences (*P* < 0.05).

## Results

### Expression of LAD1 transcript correlates with poor prognosis of colorectal cancer

**Fig. 1:**
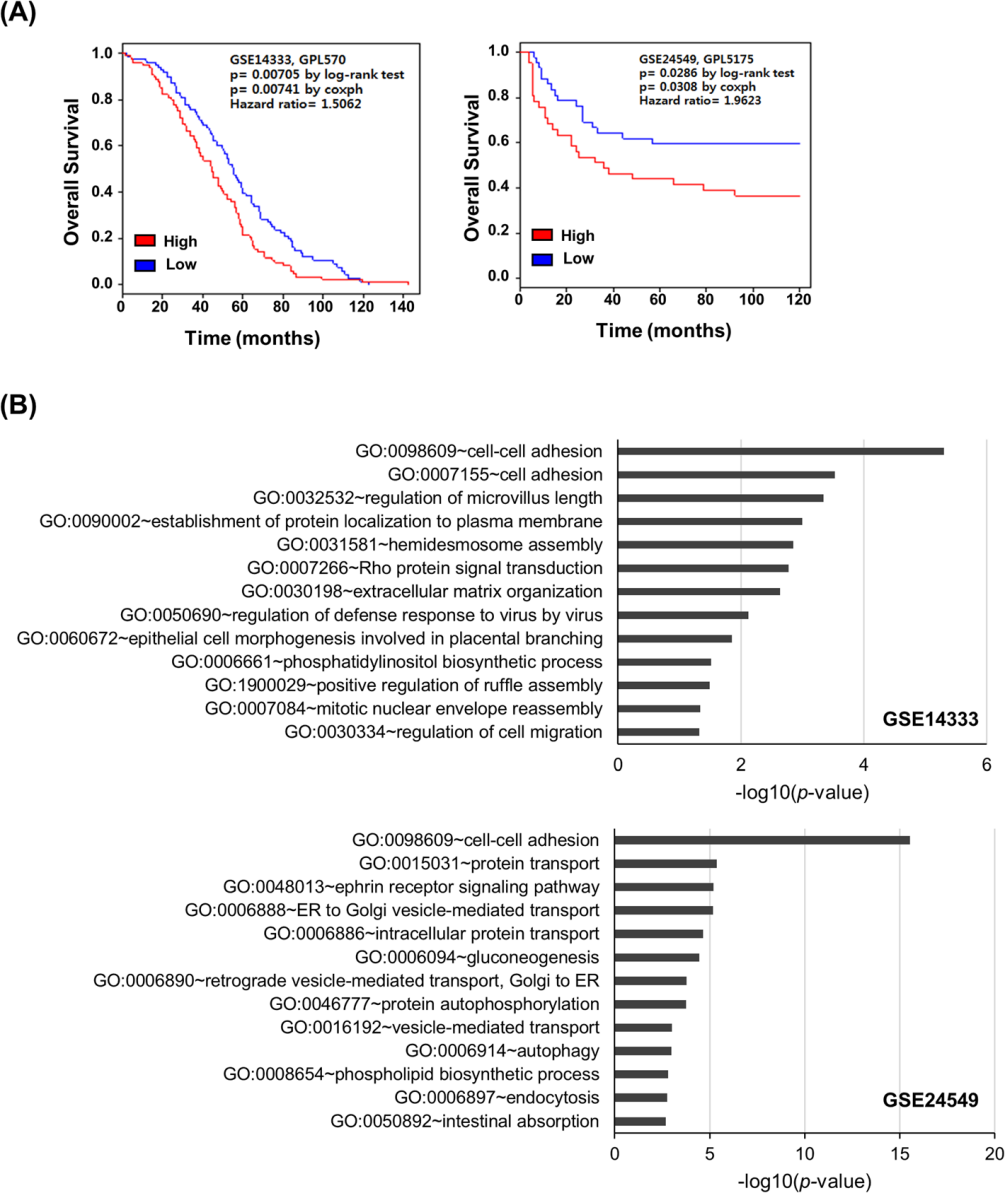
LAD1 expression is correlated with poor prognosis in colorectal cancer patients. **a** Patients with high LAD1 expression exhibited poor overall survival. The median expression value was used for stratification of patients into the high LAD1 expression (red) and the low LAD1 expression (blue) groups. Kaplan-Meier plots show significant correlations of LAD1 expression with poor survival of colorectal cancer patients whose gene expression data were extracted from GSE14333 (left) and GSE24549 (right). **b** Biological process annotation chart of genes that positively correlated with LAD1 in GSE14333 (upper) and GSE24549 (lower). Gene Ontology of the genes whose expression was positively correlated with LAD1 expression was analyzed by DAVID [16]

To determine the clinical relevance of LAD1 to colorectal cancer, we assessed the correlation of LAD1 mRNA levels with the clinical outcome of colorectal cancer patients. Analyses of two independent public datasets (GSE14333 and GSE24549) [14, 15] showed that the overall survival rate of patient groups with high LAD1 expression was significantly lower than that of the low LAD1 expression groups (Fig. 1a). The proportion of patients who survived at least 10 years was ~ 50% in GSE24549, whereas only 0.9% of patients in GSE14333 survived in 10 years. Despite the difference in cohort compositions, the hazard ratios of LAD1 expression in GSE14333 and GSE24549 were both larger than 1.0: 1.5062 and 1.9623 in GSE14333 and GSE24549, respectively. This finding indicates that LAD1 expression correlates with poor prognosis of colorectal cancer. To gain a better understanding of the function of LAD1 in colorectal cancer, we investigated genes in GSE14333 and GSE24549 whose expression was positively correlated with LAD1 expression (R > 0.4, *p* < 0.001). Gene Ontology analyses revealed that biological processes involved in cell morphology and motility, such as cell-cell adhesion and regulation of cell migration, were significantly correlated with LAD1 expression (Fig. 1b).

### LAD1 is involved in the metastatic motility of colorectal cancer cells in vitro

**Fig. 2:**
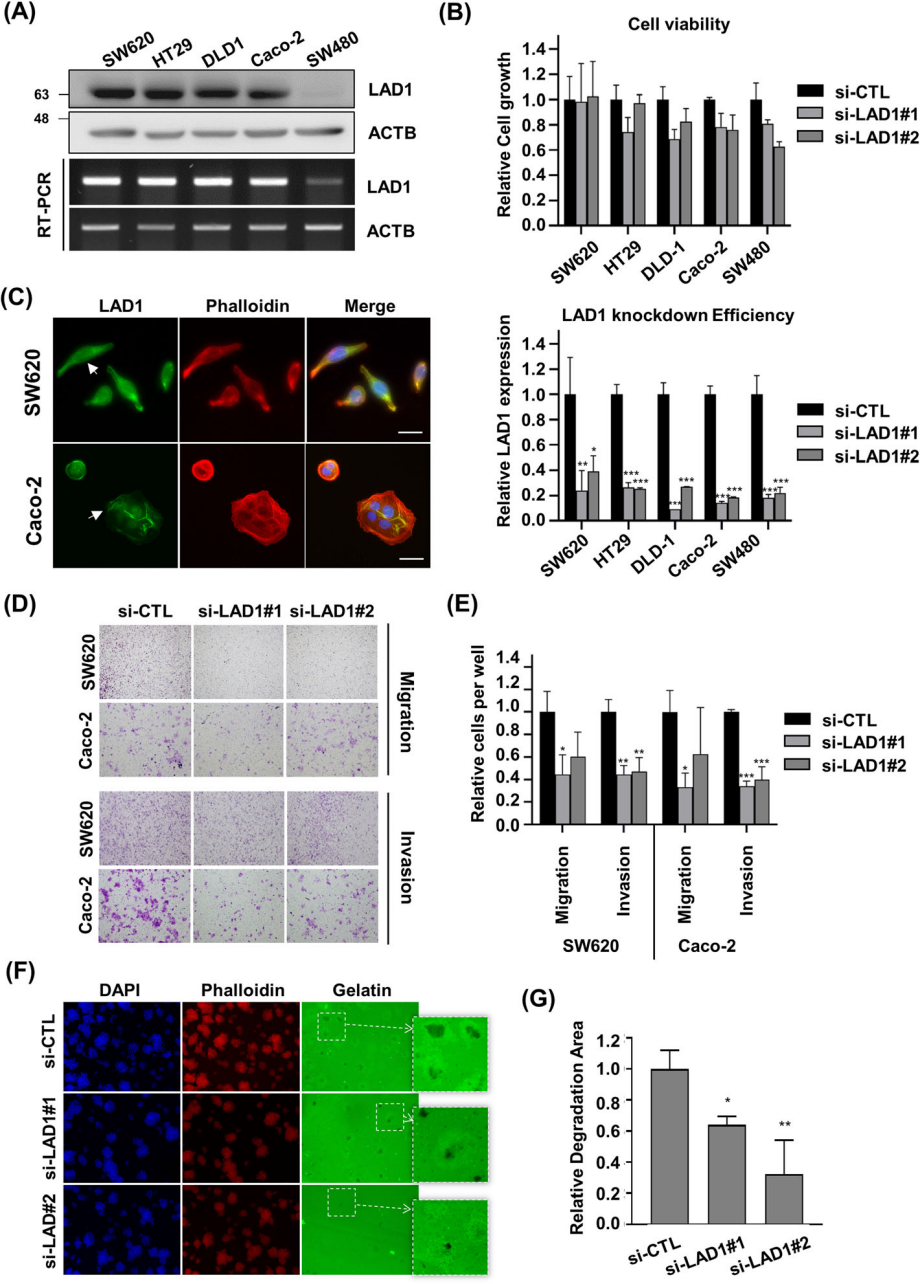
LAD1 is involved in the migration and invasion of colorectal cancer cells in vitro. **a** LAD1 was highly expressed in colorectal cancer cell lines. The expression of LAD1 in diverse human colorectal cancer cell lines was determined by immunoblots (IB) with anti-LAD1 antibody and RT-PCR. Shown are the cropped blot and gel images. **b** LAD1 depletion had little effect on the viability of colorectal cancer cells in vitro. The viability of colorectal cancer cells was examined with a Cell Titer-Blue Cell® viability assay. Relative cell growth was calculated by normalizing fluorescent signals in si-LAD1 with those in si-CTL (upper). The knockdown efficiency of LAD1 in human colorectal cancer cell lines was estimated by qRT-PCR (lower). Error bars represent the mean standard deviation (*n* = 3; *, *p* < 0.05, **, *p* < 0.01, ***, *p* < 0.001). **c** LAD1 was detected at cellular edges and partly with actin filaments in SW620 and Caco-2 cells. Localization of LAD1 and F-actin was visualized by immunofluorescence using anti-LAD1 antibody (green) and phalloidin (red). Scale bars denote 20 μm. Arrows indicate stress fiber-like structures stained by LAD1. **d** and **e** LAD1 knockdown inhibits the migration and invasion of SW620 and Caco-2 cells. SW620 and Caco-2 cells depleted of LAD1 with siRNAs were subjected to Transwell assays with or without Matrigel coating for migration or invasion measurements. Representative images of migrated and invaded cells are shown in (**d**). Relative cell numbers of migrated and invaded cells per well were normalized by the numbers of si-CTL-treated cells with those of si-LAD1-treated cells (**e**). Error bars represent the mean standard deviation (*n* = 3; *, *p* < 0.05, **, *p* < 0.01, ***, *p* < 0.001) (**f** and **g**) LAD1 depletion reduces the invadopodia-forming capability of SW620 cells. Degraded areas of fluorescently labeled gelatin matrix were visualized (**f**), and fluorescent-free areas were measured by ImageJ. Relative degradation area was calculated by normalizing fluorescent-free areas in si-LAD1 cells with those in si-CTL cells (**g**). Error bars represent the mean standard deviation (*n* = 3; *, *p* < 0.05, **, *p* < 0.01)

**Fig. 3:**
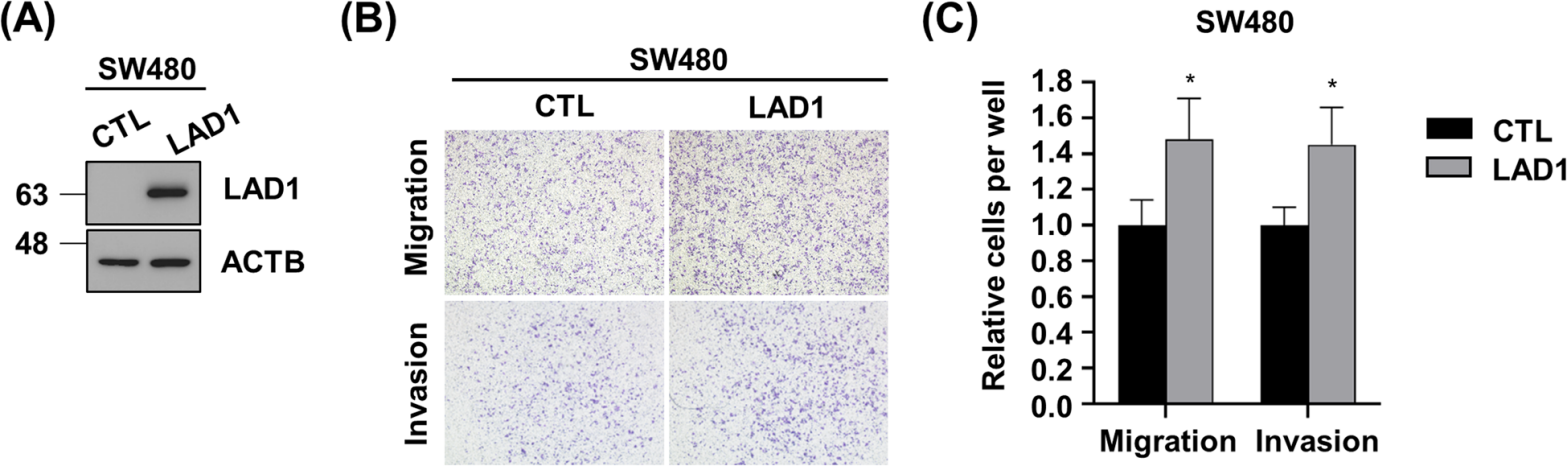
Overexpression of ectopic LAD1 promotes migration and invasion of SW480 cells in vitro. **a** LAD1 expression increased in SW480 cells transfected with ectopic LAD1 (LAD1) compared with counterpart cells with mock vector (CTL). **b** and **c** LAD1 overexpression increased the migration and invasion of SW480 cells determined by Transwell assays. Representative images of migrated and invaded cells are shown in (**b**). Relative cell numbers of migrated and invaded cells per well were normalized by the numbers of CTL cells with those of LAD1 cells (**c**). Error bars represent the mean standard deviation (*n* = 3; *, *p* < 0.05)

We detected substantial expression of LAD1 in different colorectal cancer cell lines except SW480 (Fig. 2a). Accordingly, we decided to examine whether LAD1 expression is involved in the viability and growth of colorectal cancer cells. Knockdown (KD) of LAD1 by siRNAs had little effect on cell viability (Fig. 2b). This indicates that LAD1 is dispensable for colorectal cancer cell survival. Then, to gain insight into the biological function of LAD1 in colorectal cancer cells, we determined the cellular localization of LAD1. Immunostaining of SW620 and Caco-2 cells showed that LAD1 highly localized at the cellular edges (Fig. 2c). In addition, LAD1 organized actin-like bundles in the cytosol. Co-staining with phalloidin to visualize filamentous actin (F-actin) revealed that LAD1 partly colocalized with stress fibers in cells. This result raised the possibility that LAD1 might be associated with the regulation of the morphology and/or motility of colorectal cancer cells. To test this possibility, we depleted LAD1 in SW620 and Caco-2 cells, both of which exhibited high expression of LAD1, and observed no prominent morphological changes in LAD1 KD cells. In contrast, Transwell assays revealed that LAD1 KD largely impaired the migration of both SW620 and Caco-2 cells (Fig. 2d and e). Moreover, the lack of LAD1 significantly compromised the invasion capability of SW620 and Caco-2 cells, as demonstrated by Matrigel-coated Transwell assays. The finding that LAD1 depletion resulted in more significant defects in invasion rather than migration suggests that the function of LAD1 may be more intimately associated with the invasive capability of cancer cells, such as the degradation of extracellular matrix by diverse proteases. Accordingly, the assay to examine invadopodia activity by measuring the area of fluorescently labeled gelatin matrix showed that LAD1 KD decreased the label-free area around the cells, which was indicative of degradation of the extracellular matrix via active invadopodia (Fig. 2f and g). Together, these results imply that LAD1 is functionally involved in the metastatic motility of colorectal cancer cells to invade through the extracellular barrier. It is notable that overexpression of ectopic LAD1 (Fig. 3a) increased the migration and invasion of SW480 (Fig. 3b and c) in which endogenous LAD1 expression was largely lower than the other colorectal cancer cells (Fig. 2a). These findings support that LAD1 protein expression facilitates metastatic movement of colorectal cancer cells.

### LAD1 expression is dispensable for the expression of genes involved in cancer cell migration and invasion

**Fig. 4:**
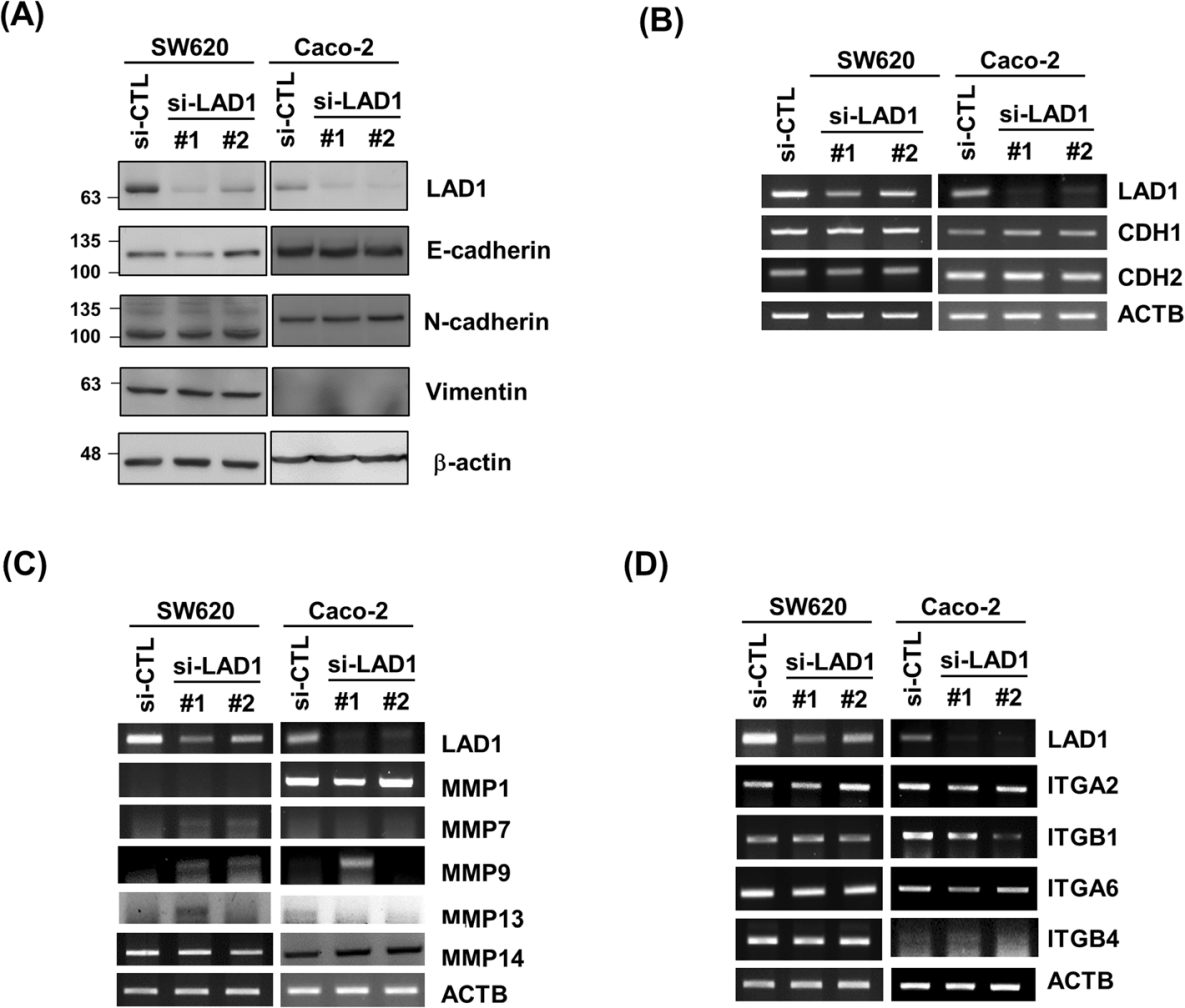
LAD1 expression barely affects the expression of EMT markers, matrix metalloproteases, and integrins. **a**, **b** LAD1 knockdown had little effect on the expression of EMT markers in SW620 and Caco-2 cells. Expression of the E-cadherin, N-cadherin, and vimentin proteins was determined by immunoblots with specific antibodies against each protein (**a**) and by RT-PCR (**b**). **c**, **d** LAD1 depletion caused little change in the expression of matrix metalloproteases (**c**) and integrins (**d**) in SW620 and Caco-2 cells. The RNA levels of each gene were measured by RT-PCR. Note that the cropped blot and gel images are shown

To explore the molecular mechanisms involved in the regulation of colorectal cancer cell motility by LAD1, we first determined the effect of LAD1 on the expression of epithelial-mesenchymal transition (EMT) markers. The protein levels of E-cadherin, N-cadherin, and vimentin, which are often used to monitor the EMT state [17], were not notably altered by LAD1 KD in SW620 and Caco-2 cells (Fig. 4a). In addition, LAD1 depletion led to no detectable change in the mRNA levels of these proteins (Fig. 4b). We then examined the expression of matrix metalloproteases (MMP1, 7, 9, 13, and 14) involved in matrix degradation [18] by measuring the mRNA levels of these genes and found little effect of LAD1 loss on their expression (Fig. 4c). In Caco-2 cells, LAD1 KD slightly reduced the mRNA expression of integrins such as ITGA2, ITGB1, and ITGA6, which are involved in cancer cell motility [19] (Fig. 4d). However, LAD1 depletion failed to decrease integrin expression in SW620 cells. Thus, it is unlikely that a compromised expression of integrins might be related to defects in LAD1-mediated invasion in both SW620 and Caco-2 cells. In summary, we propose that LAD1 hardly affects the expression of genes involved in the metastatic motility of SW620 and Caco-2 cells.

### LAD1 depletion inhibits liver metastasis of colorectal cancer cells in vivo

**Fig. 5:**
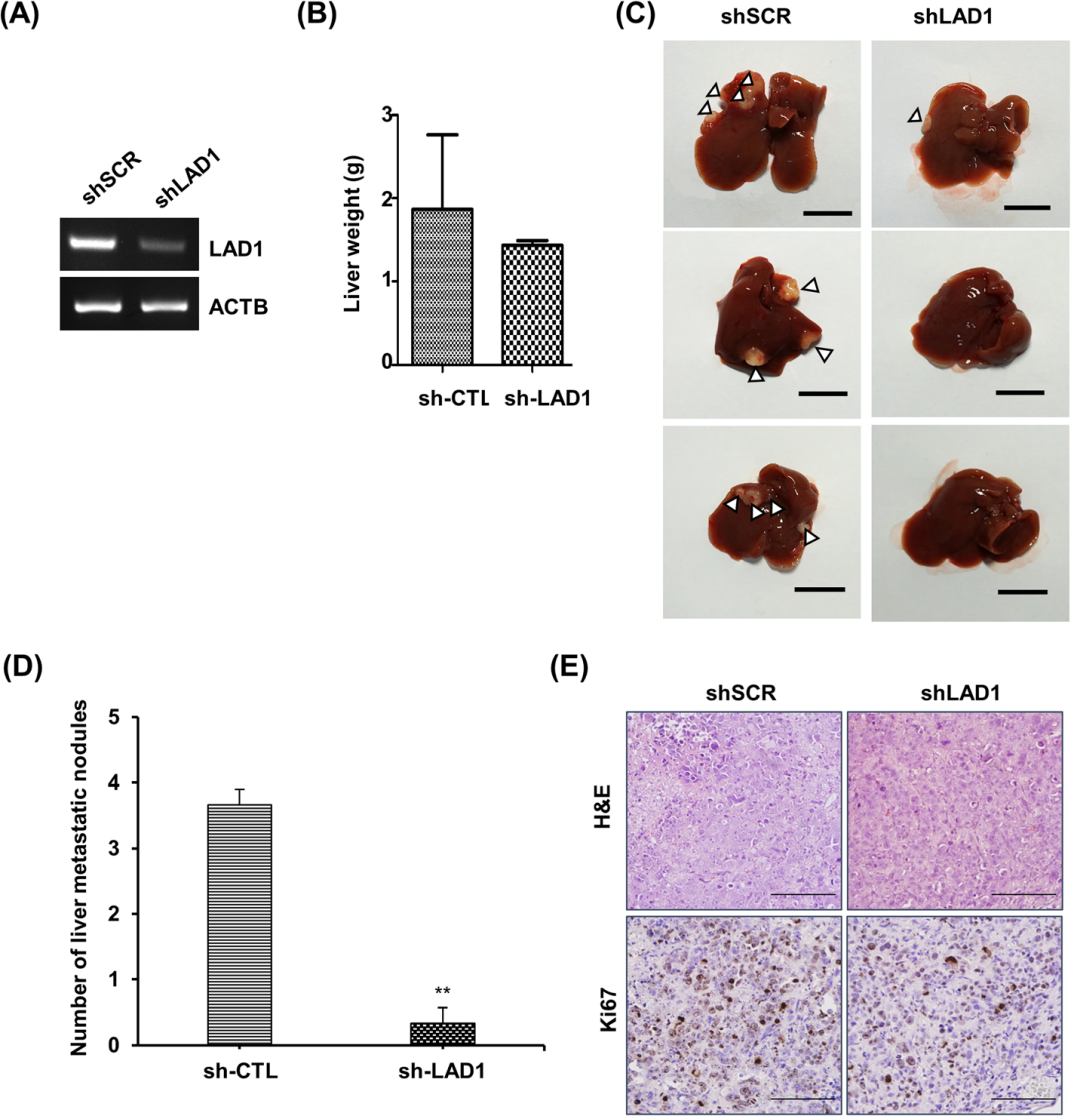
LAD1 contributes to liver metastasis of SW620 cells in vivo. **a** LAD1 in SW620 cells was stably knocked down by shRNA. The levels of LAD1 RNA in cells depleted of LAD1 (shLAD1) or control cells infected with virus carrying scramble RNA (shSCR) were determined by RT-PCR. Shown are the cropped gel image. **b** The liver weights of mice injected with SW620 cells depleted of LAD1 (shLAD1, n = 3) were comparable with those of the control (shSCR, n = 3). **c**, **d** Livers from shLAD1 mice carried fewer tumor nodules (marked by arrowheads) than those from shSCR mice. Representative images of metastatic nodules in mouse livers with scale bars for 1 cm are shown in **c**. The number of metastatic nodules in the liver is shown in (**d**). **e** H&E staining and immunohistochemistry with anti-Ki67 antibody showed that the nodules in the liver were tumorigenic. Scale bars denote 200 μm

To corroborate the functional involvement of LAD1 in colorectal cancer metastasis, we established a metastasis model in nude mice. LAD1 expression in SW620 cells was stably knocked down by shRNA (Fig. 5a). Then, we injected cells depleted of LAD1 (shLAD1) and control cells infected with nontargeting scramble shRNA (shSCR) into the spleen of nude mice. Seven weeks later, we sacrificed the mice and dissected the livers and spleens for analysis. The liver weight was comparable between shSCR- and shLAD1-injected mice (Fig. 5b). In contrast, 100% of shSCR mice (3/3) developed liver metastasis, while only 33% of shLAD1 mice (1/3) displayed tumor-like nodules in the liver (Fig. 5c). shSCR mice contained more metastatic nodules than shLAD1 mice (Fig. 5d). Furthermore, immunohistochemical analyses of liver tissues taken from the mice revealed that the intensities of H&E and Ki-67 staining in shLAD1 mice were lower than those in shSCR mice (Fig. 5e). This indicates that LAD1 depletion compromised the potential of colorectal cancer cells to metastasize for proliferation in the liver.

### Increase in LAD1 expression is associated with metastatic colorectal cancer progression

**Fig. 6:**
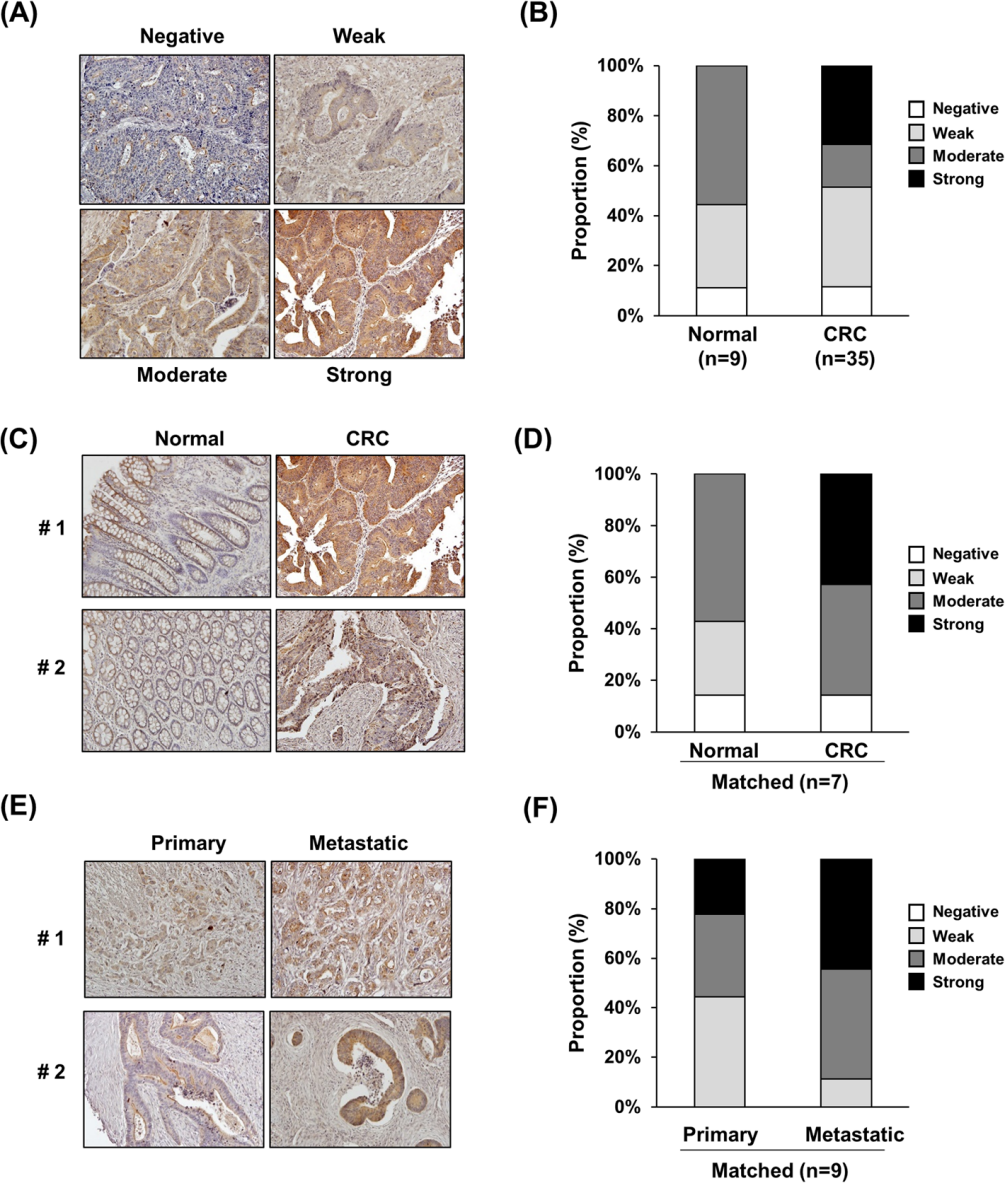
Enhanced LAD1 expression is associated with metastatic colorectal cancer tissues. **a**, **b** LAD1 expression was enriched in colorectal cancer (CRC) tissues compared with normal tissues. Staining intensities of anti-LAD1 antibody in colorectal cancer tissues were stratified into 4 categories (negative, weak, moderate, and strong; A). Strongly stained tissues were detected only in CRC (*n* = 35) and not in normal controls (*n* = 9; **b**). **c**, **d** Strong LAD1 staining was observed only in CRC tissues and not in the matched normal tissues (*n* = 7). Representative images of LAD1 immunohistochemistry and the proportion of differentially stained LAD1 tissues are shown in (**c**) and (**d**), respectively. **e**, **f** Comparison of matched primary and metastatic CRC tissues (**e**) showed a higher proportion of strong LAD1 staining intensities in metastatic tissues (*n* = 9; **f**)

To verify the association of LAD1 with the metastatic progression of colorectal cancer in humans, we examined the level of LAD1 protein in colorectal cancer tissues. Depending on the staining intensity by an anti-LAD1 antibody, the specimens were stratified into four different groups: negative, weak, moderate, and strong (Fig. 6a). Thirty-one percent of colorectal cancer tissues (11/35) exhibited strong LAD1 staining, whereas none of the normal tissues (0/9) displayed strong LAD1 signals (Fig. 6b). Comparative analysis of matched normal and cancer tissues showed a similar bias of strong LAD1 staining intensity toward colorectal cancer (0% vs 43% in normal and cancer tissues, respectively; Fig. 6c and d). Furthermore, metastatic cancer tissues tended to display elevated LAD1 staining intensities compared with matched primary cancer tissues (Fig. 6e and f). Collectively, these results suggest that the expression of LAD1 protein is positively associated with metastatic colorectal cancer progression.

## Discussion

The low survival rate of patients with regional and distant metastatic tumors compared with those with localized diseases raises the need for more extensive efforts to improve the outcomes of patients with metastasis [2]. Biomarkers related to metastatic progression facilitate efficacious clinical approaches to overcome the poor prognosis of metastatic cancer. Metastasis is a complex process involving multiple steps, such as invasion of tumor cells into the tissue surrounding the primary tumor, entry and extravasation through the bloodstream and colonization at secondary sites [2]. Tumorigenic factors in tumor cells whose functions are involved in metastatic pathways have the potential to be metastatic biomarkers. In this study, we suggest LAD1 as a positive regulator of the metastatic progression of colorectal cancer. Our findings show that (i) the expression of LAD1 transcript correlates with poor prognosis of colorectal cancer patients, (ii) depletion of LAD1 impairs the metastatic potential of colorectal cancer cells in vitro and in vivo, and (iii) elevated expression of LAD1 protein in tissues is associated with the metastatic stage of colorectal cancer.

LAD1 was originally proposed as a component of the basement membrane in mice [4]. More recently, an extensive characterization of LAD1 in human mammary epithelial cells showed that most LAD1 protein was localized in the cytosol [6]. Furthermore, the latter revealed colocalization of LAD1 with actin filament, filamins, and actin-cross-linking cytoskeletal proteins. The same study also reported that LAD1 physically interacted with stratifin (14–3-3σ), which promoted breast tumor invasion by regulating actin filament turnover [20]. We also observed intracellular localization of LAD1 partly together with filamentous actin. However, how LAD1 promotes the migration and invasion of cancer cells remains obscure. LAD1 KD failed to change the expression of genes involved in metastatic pathways such as EMT (E-cadherin and N-cadherin) [17] and matrix degradation (MMPs) [18]. In addition, LAD1-depleted cells displayed no prominent alteration in the expression of integrins that modulate signaling pathways in cancer cells [19]. Nonetheless, LAD1 loss mediated defects in invadopodia activity to degrade the extracellular matrix [21] and consequently impaired the invasion and migration of cancer cells. Considering these results, we speculate that LAD1 is unlikely to be involved in gene expression for cancer cell motility. Alternatively, based on its cellular localization, we hypothesize that LAD1 may modulate the reorganization of the cytoskeleton possibly through interaction with actin filaments and thus promote the invasive movement of cancer cells. Extensive interactome studies could help further understand the molecular mechanism of LAD1 in metastatic cancer. Although understanding the exact function of LAD1 requires additional studies in future, our findings in this study suggest that LAD1 has a potential being a prognostic biomarker for metastatic colorectal cancer.

## Conclusions

To our knowledge, this is the first study to show that LAD1 expression is associated with the metastatic motility of colorectal cancer cells in vitro and in vivo. In addition, enhanced LAD1 staining in metastatic tumor tissues suggests that LAD1 is possibly involved in the progression of metastatic colorectal cancer. Future studies exploring the molecular mechanism by which LAD1 regulates the metastatic capability of colorectal cancer cells will help to expand the repertoire of possible therapeutic approaches to overcome colorectal cancer metastasis.

## Supplementary Information

**Additional file 1: Supplemental Table 1.** RT-PCR primer list.

**Additional file 2: Supplemental Table 2.** Information of metastatsis tissue from a human colon cancer tissue array (CDA3-G, SuperBioChips).

**Additional file 3: Supplemental Table 3.** Results of short tandem repeat (STR) profiling (HPBIO, Inc., Seoul, South Korea).

## Data Availability

The datasets analyzed during the current study are available in the Gene Expression Omnibus database, GSE14333 (https://www.ncbi.nlm.nih.gov/geo/query/acc.cgi?acc=GSE14333) and GSE24549 (https://www.ncbi.nlm.nih.gov/geo/query/acc.cgi?acc=GSE24549).

## Abbreviations

LAD1: ladinin-1
METABRIC: Molecular Taxonomy of Breast Cancer International Consortium
BRAF: v-raf murine sarcoma viral oncogene homolog B1
RET: Rearranged during transfection
RAS: Rat sarcoma virus
KD: knockdown
EMT: epithelial-mesenchymal transition
MMP: matrix metalloproteinase
ITG: integrin
H&E: Hematoxylin and eosin
