# Supplementary material for: LAD1 expression is associated with the metastatic potential of colorectal cancer cells
Source: BMC Cancer. 2020 Dec 2;20:1180. doi: 10.1186/s12885-020-07660-0 (PMC7709356; doi:10.1186/s12885-020-07660-0)
Supplement: Supplementary file 1 — Additional file 1: Supplemental Table 1. RT-PCR primer list. [file 12885_2020_7660_MOESM1_ESM.docx]

**Supplemental table1**. RT-PCR primer list

| Gene | Primer sequence (5'-3') | |
| --- | --- | --- |
| LAD1 | forward | ACCTACAGCAGCTCCCTCAA |
|  | reverse | ATGGCCGTGTGGTATCTCTC |
| ACTB | forward | GGACTTCGAGCAAGAGATGG |
|  | reverse | AGCACTGTGTTGGCGTACAG |
| CDH1 | forward | TGGAACAGGGACACTTCTGC |
|  | reverse | TGGGTTGGGTCGTTGTACTG |
| CDH2 | forward | CCTTTCAAACACACTCCACGG |
|  | reverse | TGTTTGGGTCGGTCTGCTATG |
| MMP1 | forward | CTGAAGGTGATGAAGCAGCC |
|  | reverse | AGTCCAAGAGAATGGCCGAG |
| MMP7 | forward | TGGGGAACTGCTGACATCAT |
|  | reverse | CCCTAGACTGCTACCATCCG |
| MMP9 | forward | GAGTTCCCGGAGTGAGTTGA |
|  | reverse | AAAGGTGAGAAGAGAGGGCC |
| MMP13 | forward | TTGAGCTGGACTCATTGTCG |
|  | reverse | GGAGCCTCTCAGTCATGGAG |
| MMP14 | forward | CAACACTGCCTACGAGAGGA |
|  | reverse | GTTCTACCTTCAGCTTCTGG |
| ITGA2 | forward | GGGCATTGAAAACACTCGAT |
|  | reverse | TCGGATCCCAAGATTTTCTG |
| ITGB1 | forward | CATCTGCGAGTGTGGTGTCT |
|  | reverse | GGGGTAATTTGTCCCGACTT |
| ITGA6 | forward | GGAGCCCCACAGTATTTTGA |
|  | reverse | TTCCATTTGCA GATCCATGA |
| ITGB4 | forward | CCCCCTTCTCCTTCAAGAAC |
|  | reverse | GCTGACTCGGTGGAGAAGAC |
