# Supplementary material for: LAD1 expression is associated with the metastatic potential of colorectal cancer cells
Source: BMC Cancer. 2020 Dec 2;20:1180. doi: 10.1186/s12885-020-07660-0 (PMC7709356; doi:10.1186/s12885-020-07660-0)
Supplement: Supplementary file 2 — Additional file 2: Supplemental Table 2. Information of metastatsis tissue from a human colon cancer tissue array (CDA3-G, SuperBioChips). [file 12885_2020_7660_MOESM2_ESM.docx]

| No. | organ | diagnosis | diagnosis of primary tumor | Months* |
| --- | --- | --- | --- | --- |
| 1 | lung | metastatic carcinoma from #31 | adenocarcinoma, moderately differentiated | 45 |
| 2 | lymph node | metastatic carcinoma from #32 | adenocarcinoma, poorly differentiated | 0 |
| 3 | lung | metastatic carcinoma from #33 | adenocarcinoma, moderately differentiated | 17 |
| 4 | lung | metastatic carcinoma from #35 | adenocarcinoma, moderately differentiated | 36 |
| 5 | liver | metastatic carcinoma from #36 | adenocarcinoma, poorly differentiated | 0 |
| 6 | ovary | metastatic carcinoma from #37 | adenocarcinoma, moderately differentiated | 17 |
| 7 | uterus | metastatic carcinoma from #38 | adenocarcinoma, moderately differentiated | 0 |
| 8 | liver | metastatic carcinoma from #39 | adenocarcinoma, moderately differentiated | 0 |
| 9 | omentum | metastatic carcinoma from #40 | adenocarcinoma, moderately differentiated | 0 |

**Supplemental table2**. Information of metastatsis tissue from a human colon cancer tissue array (CDA3-G, SuperBioChips)

Months*: interval between primary and metastatic cancer
