# Supplementary material for: LAD1 expression is associated with the metastatic potential of colorectal cancer cells
Source: BMC Cancer. 2020 Dec 2;20:1180. doi: 10.1186/s12885-020-07660-0 (PMC7709356; doi:10.1186/s12885-020-07660-0)
Supplement: Supplementary file 3 — Additional file 3: Supplemental Table 3. Results of short tandem repeat (STR) profiling (HPBIO, Inc., Seoul, South Korea). [file 12885_2020_7660_MOESM3_ESM.docx]

**Supplemental table3**. Results of short tandem repeat (STR) profiling (HPBIO, Inc., Seoul, South Korea)

| **Cell lines** | **SW620** | | **HT-29** | | **DLD-1** | | **Caco-2** | | **SW480** | |
| --- | --- | --- | --- | --- | --- | --- | --- | --- | --- | --- |
| Locus | Allele1 | Allele2 | Allele1 | Allele2 | Allele1 | Allele2 | Allele1 | Allele2 | Allele1 | Allele2 |
| AMEL | X | | X | | XY | | X | | X | |
| CSF1PO | 13 | 14 | 11 | 12 | 11 | 12 | 11 | 11 | 13 | 14 |
| D13S317 | 12 | 12 | 11 | 11 | 8 | 11 | 11 | 13/14 | 12 | 12 |
| D16S539 | 9 | 13 | 11 | 12 | 12 | 13 | 12 | 13 | 13 | 13 |
| D5S818 | 13 | 13 | 11 | 12 | 13 | 13 | 12 | 13 | 13 | 13 |
| D7S820 | 8 | 9 | 10 | 10 | 10 | 12 | 11 | 12 | 8 | 8 |
| TH01 | 8 | 8 | 6 | 9 | 7 | 9.3 | 6 | 6 | 8 | 8 |
| TPOX | 11 | 11 | 8 | 9 | 8 | 11 | 9 | 11 | 11 | 11 |
| vWA | 16 | 16 | 17 | 19 | 18 | 19 | 16 | 18 | 16 | 16 |
